# Prediction of procedural success of transcatheter mitral valve repair with normal and extended clip arms

**DOI:** 10.1007/s10554-021-02514-7

**Published:** 2022-01-04

**Authors:** Annemarie Kirschfink, Mhd Nawar Alachkar, Mohammad Almalla, Julian Grebe, Felix Vogt, Jörg Schröder, Michael Frick, Nikolaus Marx, Ertunc Altiok

**Affiliations:** Department of Cardiology, Angiology and Intensive Care, Medical Clinic I, University Hospital, RWTH Aachen University, Pauwelsstrasse 30, 52074 Aachen, Germany

**Keywords:** 3-dimensional echocardiography, Transesophageal echocardiography, Secondary mitral regurgitation, Transcatheter mitral valve repair

## Abstract

TMVR using different clip sizes is a treatment option for selected patients with mitral regurgitation (MR). This study sought to identify predictors of successful transcatheter mitral valve repair (TMVR) by 3-dimensional (3D) echocardiography and to compare different effects of the larger XTR and the smaller NT/NTR devices. 3D transesophageal echocardiography was performed on 54 patients with secondary MR undergoing TMVR with one clip (55.6% NT/NTR, 44.4% XTR). All NT/NTR and 96% of XTR patients had MR reduction ≤ 2+. Despite more severe baseline MR (3D vena contracta area (VCA): 0.67 ± 0.34 cm^2^ vs. 0.43 ± 0.19 cm^2^, p = 0.004) and greater mitral valve area (MVA) (6.8 ± 2.1 cm^2^ vs. 5.1 ± 1.6 cm^2^, p = 0.001) in the XTR group, MR severity after TMVR was not different between XTR and NT/NTR patients (3D VCA: 0.19 ± 0.14 vs. 0.17 ± 0.10, p = 0.51). Baseline 3D VCA > 0.45 cm^2^ in NT/NTR (AUC = 0.802, 95% CI 0.602 to 1.000) and 3D VCA > 0.54 cm^2^ in XTR devices (AUC = 0.868, 95% CI 0.719 to 1.000) were associated with ineffective MR reduction defined as residual VCA ≤ 0.2 cm^2^. Baseline MVA ≤ 4.2 cm^2^ in NT/NTR (AUC = 0.920, 95% CI 0.809 to 1.000) and MVA ≤ 6.0 cm^2^ in XTR devices (AUC = 0.865, 95% CI 0.664 to 1.000) were associated with postprocedural transmitral pressure gradient (TMPG) ≥ 5 mmHg. TMVR using the XTR device resulted in an equally effective reduction of MR despite of a greater baseline MR. Distinct cut-off values of baseline 3D VCA and MVA for prediction of successful MR reduction and postprocedural increase of TMPG were identified for the different devices.

## Introduction

Transcutaneous mitral valve repair (TMVR) using the edge-to-edge technique has become a well-established procedure, which is a safe treatment option for patients with symptomatic mitral regurgitation (MR) regarding specific indications [1]. TMVR with the MitraClip™ device is associated with improvements in patient symptoms and reduction of hospitalization for heart failure [2, 3]. Currently different randomized controlled clinical trials with contradictory results exist concerning its efficacy, which are in part explained by different patient characteristics especially concerning left ventricular (LV) dilatation, MR severity and guideline-directed management and therapy [3, 4]. In the past few years new clip iterations have been developed: the MitraClip™ NT Clip Delivery System was replaced by the newer-generation MitraClip™ NTR, which kept the original NT clip size, and the XTR Clip Delivery System, which features 3 mm longer clip arms and grippers to improve leaflet grasping with unchanged clip width in contrast to the next-generation MitraClip G4 system and the PASCAL device (Edwards Lifesciences, Irvine, CA, USA) [5–7].

The objective of this study was to evaluate the effects of the larger XTR and the smaller NT or NTR devices on procedural reduction of MR and to identify predictors of procedural success of TMVR using 3-dimensional (3D) echocardiography for detailed analysis.

## Methods

Into this monocentric study 54 high-risk surgical patients with moderate to severe and severe symptomatic secondary MR according to the current guidelines of the European Society of Cardiology undergoing TMVR were included [8]. Only patients who underwent TMVR with one MitraClip™ device (Abbott Vascular Structural Heart, Menlo Park, CA, USA) were included. According to their expert judgment the operators defined the clip strategy. Transcatheter treatment was confirmed by a multidisciplinary team in advance. Mean age of the population was 74.6 ± 7.5 years with a larger portion of men (61.1%). In 25 of these 54 patients (46.3%) the smaller NT, in 5 patients (9.3%) the smaller NTR and in 24 patients (44.4%) the larger XTR MitraClip device was implanted. 2-dimensional (2D) transthoracic echocardiography (TTE) was performed at baseline and before discharge as well as preprocedural and periprocedural 3D transesophageal echocardiography (TEE). All baseline patient and echocardiographic characteristics are given in Table 1. Regurgitation severity was classified by different MR grades (0 none; 1+ mild; 2+ moderate; 3+ mild-to-severe; 4+ severe) as assessed by 2D echocardiography using an integrative approach of different parameters [3]. This study was approved by the local ethics committee.

**Table 1:** Baseline patient and echocardiographic characteristics

Variable	All (n = 54)	NT/NTR (n = 30)	XTR (n = 24)	p value
Age, years	74.6 ± 7.5	72.8 ± 7.5	76.8 ± 7.0	0.05
Men, n	33	15	18	0.09
Logistic EuroSCORE, %	18.7 ± 12.7	15.4 ± 9.7	21.9 ± 15.2	0.08
Atrial fibrillation, n	35	16	19	0.08
NYHA functional classification, n				0.09
II	5 (9%)	1 (3%)	4 (17%)	
III	33 (61%)	18 (60%)	15 (63%)	
IV	16 (30%)	11 (37%)	5 (21%)	
Ejection fraction, %	40.0 ± 13.3	39.0 ± 12.3	41.1 ± 3.0	0.56
Left ventricular enddiastolic diameter (LVEDD), mm	56.7 ± 8.7	57.7 ± 7.3	55.4 ± 5.6	0.22
Left ventricular endsystolic diameter (LVESD), mm	45.8 ± 9.6	47.0 ± 9.5	43.8 ± 9.6	0.22
Mitral regurgitation severity, n				**0.002**
3+	35 (65%)	25 (83%)	10 (42%)	
4+	19 (35%)	5 (17%)	14 (58.%)	
Effective regurgitant orifice area (EROA) by 2D PISA method, cm^2^	0.30 ± 0.12	0.27 ± 0.12	0.34 ± 0.12	**0.02**
Regurgitant volume (RVol) by 2D PISA method, ml/beat	45.6 ± 18.0	44.1 ± 18.4	49.8 ± 17.4	0.25
Mitral valve opening area (MVA), cm^2^	5.9 ± 2.0	5.1 ± 1.6	6.8 ± 2.1	**0.001**
Mean transmitral pressure gradient (TMPG), mmHg	1.9 ± 0.9	1.7 ± 0.9	2.0 ± 1.0	0.36
3D VCA (3D TEE direct planimetry), cm^2^	0.54 ± 0.29	0.43 ± 0.19	0.67 ± 0.34	**0.004**
3D RVol (3D TEE VCA method), ml/beat	84.6 ± 44.8	72.4 ± 31.8	99.9 ± 53.9	**0.03**
Main zone of mitral regurgitation, n				0.90
Zone 1	1 (2%)	0	1 (2%)	
Zone 2	50 (93%)	29 (97%)	22 (92%)	
Zone 3	3 (6%)	1 (3%)	2 (8%)	
Clip position, n				0.90
Zone 1	1 (2%)	0	1 (2%)	
Zone 2	50 (93%)	29 (97%)	22 (92%)	
Zone 3	3 (6%)	1 (3%)	2 (8%)	
Tenting height, mm	7.6 ± 3.9	8.5 ± 3.8	6.3 ± 3.9	**0.04**
Coaptation length, mm	2.2 ± 1.3	2.3 ± 1.1	2.2 ± 1.4	0.70
Coaptation gap, mm	0.3 ± 1.3	0.2 ± 1.1	0.5 ± 1.5	0.33
Mitral leaflet length pre-implantation, mm				
Anterior mitral leaflet	25.2 ± 4.3	25.4 ± 4.4	23.7 ± 3.7	**0.019**
Posterior mitral leaflet	12.3 ± 2.8	12.2 ± 2.4	12.4 ± 3.3	0.75

P values less than 0.05 indicate significant differences of variables between NT/NTR and XTR group

*VCA* vena contracta area, *3D* 3-dimensional, *MVA* mitral valve area, *NYHA* New York Heart Association, *PISA* proximal isovelocity surface area, *RVol* regurgitant volume, *TEE* transesophageal echocardiography

### Image acquisition

TTE studies were performed with commercially available echocardiographic systems (Vivid E95, General Electric Vingmed, Horten, Norway) with a 2D transthoracic probe (M5S, General Electric Vingmed, Horten, Norway) for analysis of baseline echocardiographic parameters. TEE studies were performed with commercially available echocardiographic systems (EPIQ; Philips Medical Systems, Andover, MA) and a 3D transesophageal probe (X7-2t; Philips Medical Systems, Andover, MA, USA) for advanced analysis. TEE studies included standard acquisitions for 2D analysis and additional 3D acquisitions without and with color Doppler flow imaging of the mitral valve. 3D data was analyzed off-line with dedicated software (QLAB; Philips Medical Systems, Andover, MA).

### Echocardiographic analysis

Baseline echocardiographic parameters were assessed by 2D TTE according to recommendations of the American Society of Echocardiography and the European Association of Cardiovascular Imaging [9]. Chamber sizes were measured and left ventricular (LV) function was determined in transthoracic 2- and 4-chamber views by manual tracing of endocardial contours using Simpson’s biplane rule.

**Fig. 1:**
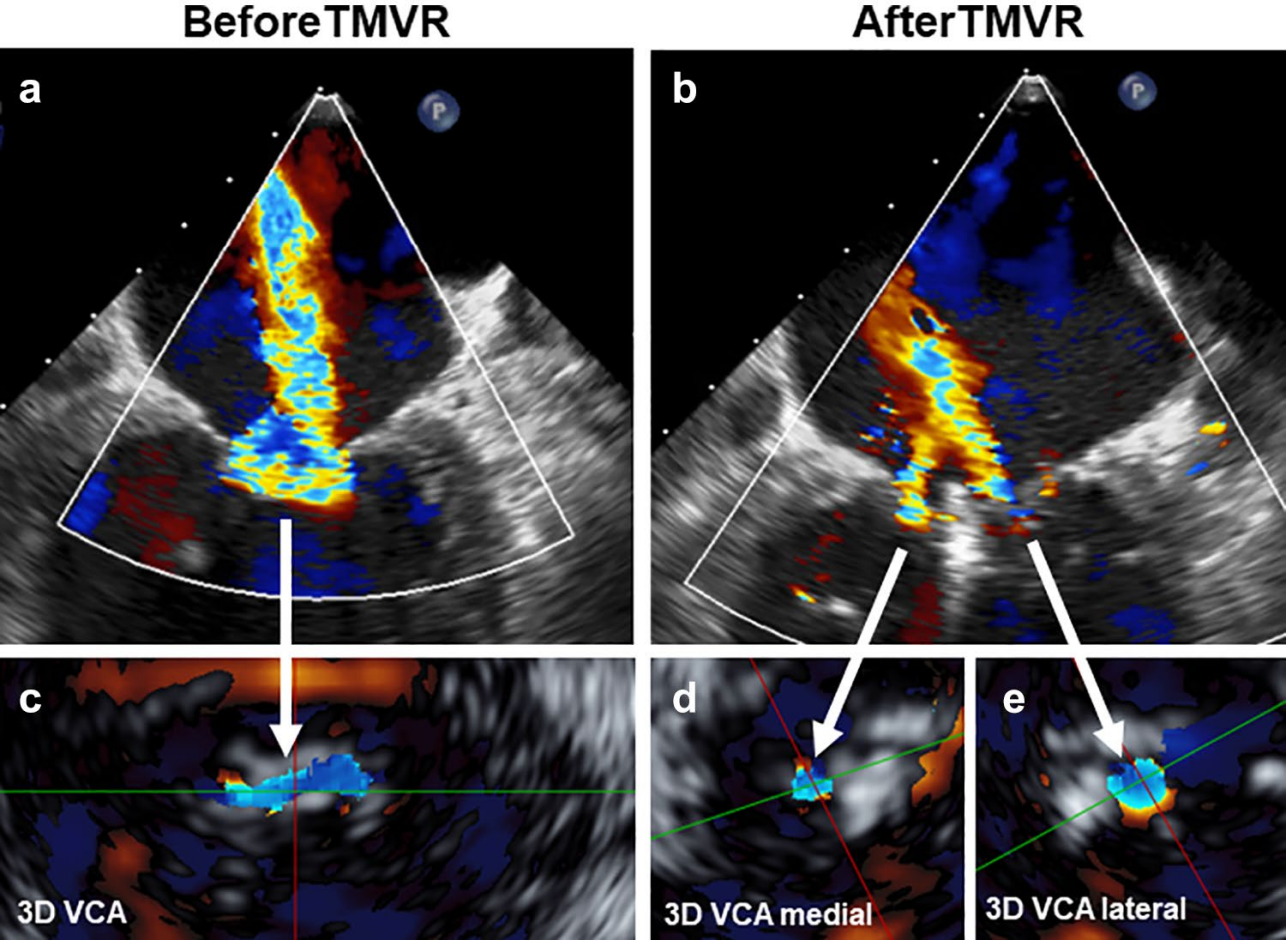
Assessment of 3D Vena contracta area (VCA). 2-dimensional TEE color flow Doppler intercommissural view before TMVR (**a**) and after deployment of one MitraClip XTR device (**b**). 3D TEE color Doppler with cut plane orthogonal to the regurgitant jet allows direct visualization of VCA before TMVR (**c**). After TMVR, 3D VCA was assessed separately for the remaining regurgitation jet located medial (**d**) and lateral to the clip (**e**). Total VCA was obtained by summation of both areas. *3D* 3-dimensional, *TEE* transesophageal echocardiography, *TMVR* transcatheter mitral valve repair, *VCA* vena contracta area

**Fig. 2:**
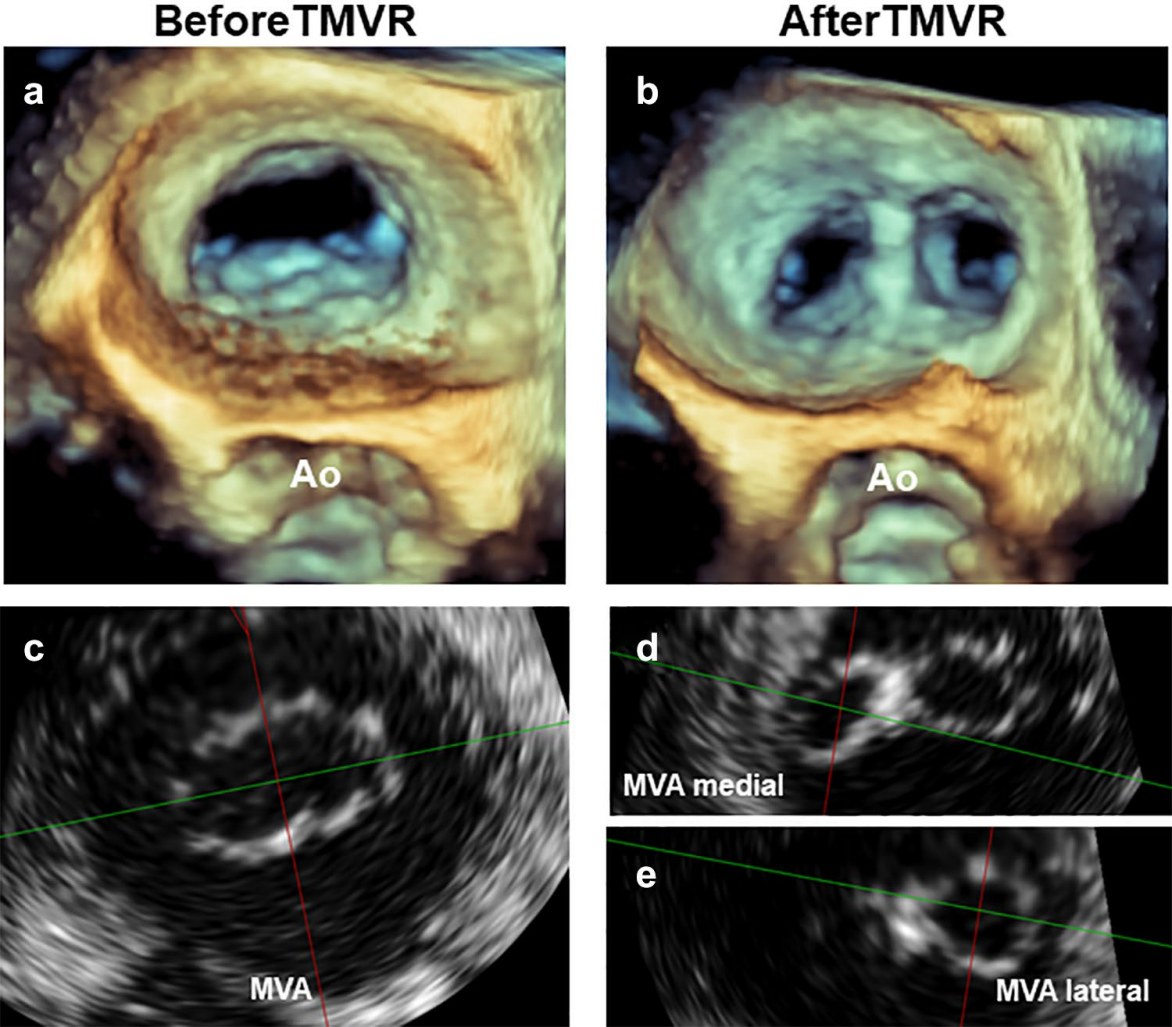
Measurements of mitral valve opening area (MVA) before and after transcatheter mitral valve repair (TMVR). 3D TEE view from the roof of the left atrium in diastole before (**a**) and after (**b**) TMVR with direct visualization of the maximum diastolic mitral valve opening area (MVA) before TMVR (**c**) and the medial and lateral MVA separately after TMVR (**d** + **e**). *3D* 3-dimensional, *MVA* indicates mitral valve opening area, *TEE* transesophageal echocardiography, *TMVR* transcatheter mitral valve repair

Effective regurgitant orifice area (EROA) of MR was calculated using the proximal isovelocity surface area method (PISA) in 2D TEE images. For calculation of the regurgitant volume (RVol), EROA and velocity time integral obtained by continuous-wave Doppler were multiplied. In 3D color Doppler TEE datasets vena contracta area (VCA) was assessed to define MR severity before and after TMVR by direct planimetry of the vena contracta (Fig. 1). For calculation of the mitral regurgitant volume (RVol), velocity time integral obtained by continuous-wave Doppler and VCA by 3D assessment before TMVR as well as lateral and medial VCA after TMVR were multiplied. For assessment of valve stenosis, baseline MVA was assessed at time of maximal diastolic opening by 3D TEE direct planimetry (Fig. 2a, c). The implantation of a clip leads to a mitral valve with a double-orifice, where the lateral and medial orifices are not in one plane any more. Therefore, after TMVR adjustment of the lateral und medial orifice were performed separately and total MVA was calculated by summing up the medial und lateral orifice area (Fig. 2b, d, e). Mean diastolic transmitral pressure gradient (TMPG) before and after TMVR was measured by continuous-wave Doppler.

### Statistical analysis

Statistical analysis was performed with the MedCalc software (Version 13.0.0.0; Mariakerke, Belgium). Continuous data are presented as mean ± standard deviation and compared with paired Student t test or independent samples t test and one-way analysis of variance (ANOVA) with Student–Newman–Keuls test for all pairwise comparisons as adequate. Categorical variables are summarized as frequencies and were assessed by Fisher’s exact test and Mann–Whitney test as adequate. Clustered multiple variables graphs with plot of all data and horizontal lines for mean with 95% confidence-interval (95% CI) was used for comparison of preprocedural parameters with postprocedural results. Receiver-operating characteristics (ROC) curve analysis with calculation of area under the curve (AUC) and according 95% CI was performed to define the impact of baseline 3D VCA on procedural reduction of MR as well as the impact of baseline MVA and baseline mean TMPG on development of valve stenosis after TMVR. Sensitivity and specificity of cut-off values with the highest Youden-index are presented. P values less than 0.05 were considered significant.

## Results

**Fig. 3:**
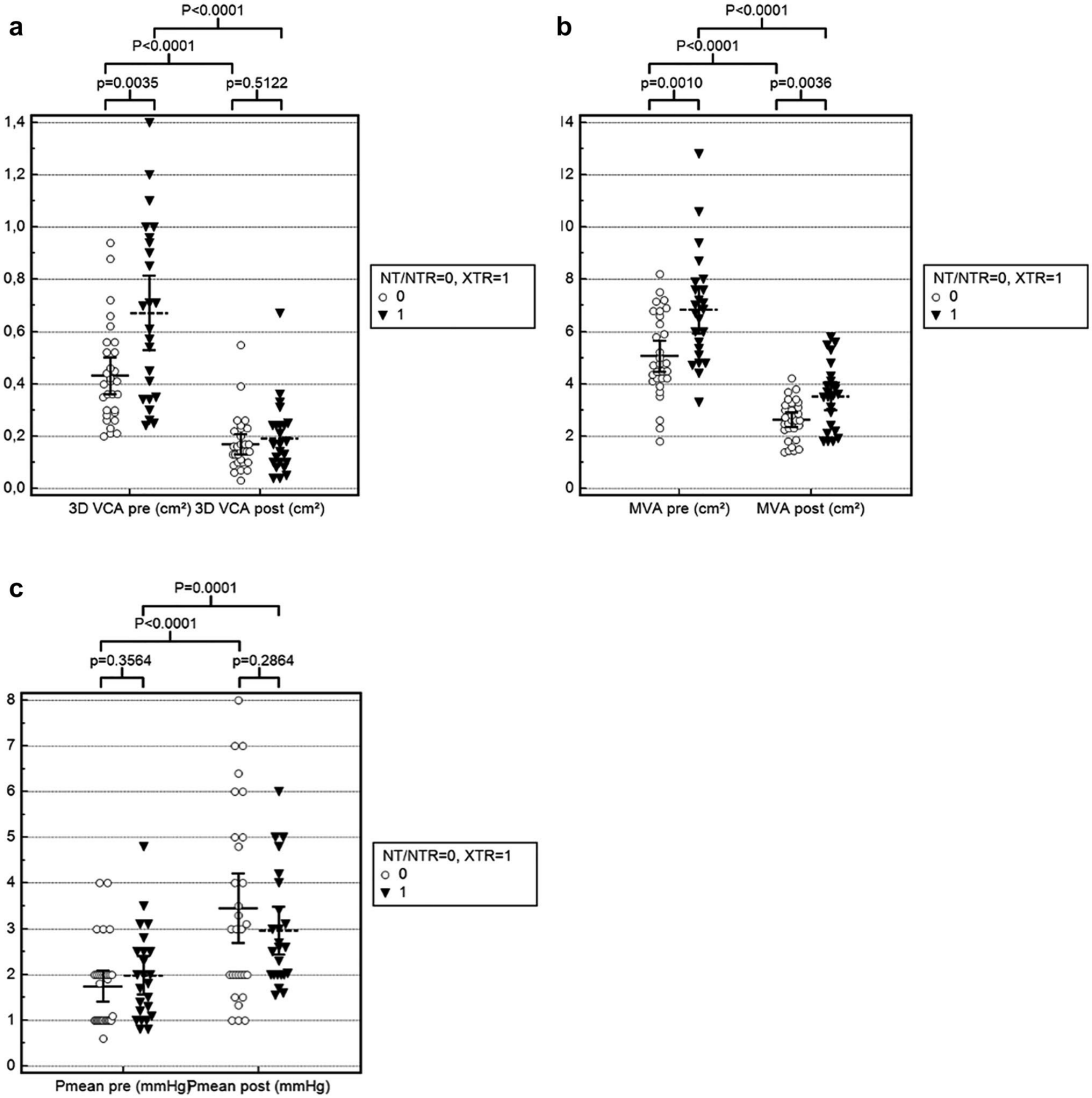
Comparison of preprocedural and postprocedural mitral regurgitation and mitral stenosis. Clustered multiple variables graphs with plot of all data for comparison of preprocedural and postprocedural measurements after TMVR of 3D VCA (**a**) and diastolic MVA (**b**) assessed by 3D TEE as well as the TMPG assessed by continuous-wave Doppler (**c**) differentiated between the NT or NTR device and the XTR device. *3D* 3-dimensional, *MVA* mitral valve opening area, *TEE* transesophageal echocardiography, *TMPG* mean transmitral pressure gradient, *VCA* vena contracta area

In this retrospective study a total of 54 patients who underwent TMVR with implantation of only one clip were analyzed using the smaller MitraClip NT/NTR device in 30 patients (55.6%) and the lager MitraClip XTR in 24 patients (44.4%). In 50 patients (93%) the clip was positioned in zone 2, in 1 patient (2%) in zone 1 and in 3 patients (6%) in zone 3 of the mitral valve. Table 1 demonstrates the baseline patients and echocardiographic characteristics. Patients treated with the XTR device had a more severe baseline MR than patients treated with the NT/NTR device: EROA by 2D PISA method was greater (0.34 ± 0.12 cm^2^ vs. 0.27 ± 0.12 cm^2^, p = 0.0249) in XTR patients as well as the 3D VCA by 3D TEE direct planimetry (0.67 ± 0.34 cm^2^ vs. 0.43 ± 0.19 cm^2^, p = 0.0035). Baseline MVA was also greater in patients treated with the XTR compared with the NT/NTR device (6.8 ± 2.1 cm^2^ vs. 5.1 ± 1.6 cm^2^, p = 0.0010) while there was no difference in baseline TMPG between both groups (2.0 ± 1.0 mmHg vs. 1.7 ± 0.9 mmHg, p = 0.36). Baseline length of anterior mitral leaflet (AML) was shorter in XTR than NTR patients (23.7 ± 3.7 mm vs. 25.4 ± 4.4 mm, p = 0.019) while posterior mitral leaflet (PML) length was not different (12.4 ± 3.3 mm vs. 12.1 ± 2.1 mm, p = 0.79). Figure 3 demonstrates the effects of TMVR from baseline to postprocedural values of the 3D VCA, MVA and TMPG differentiated between the NT/NTR and XTR device.

### Comparison of procedural effects differentiated between NT/NTR and XTR device

In all NT/NTR patients and 96% of XTR patients, a MR reduction to mild-to moderate (2 +) or less could be achieved according to 2D Doppler echocardiographic parameters. By 3D echocardiographic assessment, in all patients a significant reduction of MR as well as MVA was seen after TMVR with one clip (Table 2). Relative reduction of MR severity was equally effective by the XTR device compared to the NT/NTR device with a trend to in favor of the XTR device (Δ-VCA − 69.4 ± 16.3% vs. − 60.6 ± 15.7%, p = 0.05). Relative reduction of AML length was not different by XTR and NT/NTR device (Δ-AML length − 22.9 ± 8% vs. − 24.0 ± 8.9%, p = 0.10) while relative reduction of PML length was greater by XTR compared to NT/NTR device (Δ-PML length − 42.9 ± 11.9% vs. − 32.7 ± 8.5%, p = 0.001). However, there was no significant difference in relative reduction of MVA (Δ-MVA: − 47.9 ± 13.1% vs. − 46.0 ± 13.0%, p = 0.61) (Table 3). The increase of TMPG was not significantly different between the groups (+ 70.4 ± 70.8% by XTR vs. + 100.6 ± 75.9% by NT/NTR device, p = 0.14).

**Table 2:** Comparison of echocardiographic parameters between the NT/NTR and the XTR device

	All			NT/NTR			XTR		
	Before TMVR	After TMVR	p value	Before TMVR	After TMVR	p value	Before TMVR	After TMVR	p value
3D TEE VCA^a^, cm^2^	0.54 ± 0.29	0.18 ± 0.12	< 0.0001	0.43 ± 0.19	0.17 ± 0.10	< 0.0001	0.67 ± 0.34	0.19 ± 0.14	< 0.0001
3D TEE RVol^b^, ml/beat	84.6 ± 44.8	27.1 ± 20.6	< 0.0001	72.4 ± 31.8	25.2 ± 14.0	< 0.0001	99.9 ± 53.9	28.2 ± 27.1	< 0.0001
MVA, cm^2^	5.9 ± 2.0	3.0 ± 1.1	< 0.0001	5.1 ± 1.6	2.6 ± 0.8	< 0.0001	6.8 ± 2.1	3.5 ± 1.3	< 0.0001
TMPG, mmHg	1.9 ± 0.9	3.2 ± 1.7	< 0.0001	1.7 ± 0.9	3.4 ± 2.0	< 0.0001	2.0 ± 1.0	3.0 ± 1.2	0.0001

*3D* 3-dimensional, *MV* mitral valve, *MVA* mitral valve area, *TMPG* mean transmitral pressure gradient, *RVol* regurgitant volume, *TEE* transesophageal echocardiography, *TMVR* transcatheter mitral valve repair, *VCA* vena contracta area

^a^Assessed by direct planimetry

^b^Calculated by 3D VCA method

**Table 3:** Impact of transcatheter mitral valve repair (TMVR) on reduction of mitral regurgitation severity and changes of mitral valvular parameters

	All (n = 54)	NT/NTR (n = 30)	XTR (n = 24)	p value
Reduction of 3D VCA, %	− 64.5 ± 16.4	− 60.6 ± 15.7	− 69.4 ± 16.3	0.05
Reduction of RVol by 3D VCA method, %	− 66.2 ± 19.3	− 62.9 ± 16.3	− 70.5 ± 22.2	0.15
Reduction of MVA, %	− 46.8 ± 12.9	− 46.0 ± 13.0	− 47.9 ± 13.1	0.61

*3D* 3-dimensional, *MVA* mitral valve opening area, *RVol* regurgitant volume, *TMVR* transcatheter mitral valve repair, *VCA* vena contracta area

### Prediction of successful TMVR based on baseline echocardiographic parameters

Procedural success after TMVR was defined as reduction of MR with VCA ≤ 0.2 cm^2^ whereas unfavorable result was defined as residual VCA > 0.2 cm^2^ after the procedure. Baseline 3D VCA assessed by 3D-TEE showed to be a predictor for ineffective MR reduction with different cut-off values for the NT/NTR device (cut-off: 3D VCA > 0.45 cm^2^: AUC = 0.802, 95% CI 0.602 to 1.000; sensitivity 77.8%, specificity 85.7%) and the XTR device (cut-off: 3D VCA > 0.54 cm^2^: AUC = 0.868, 95% CI 0.719 to 1.000; sensitivity 100%, specificity 71.4%) (Table 4).

**Table 4:** Impact of baseline 3D vena contracta area (VCA) on postprocedural mitral regurgitation

	3D VCA ≤ 0.2 cm^2^ after TMVR	3D VCA > 0.2 cm^2^ after TMVR	p value	AUC (95%-CI)	Cut-off value	Sensitivity; specificity	p value
Baseline 3D VCA, cm^2^; all patients (n = 54)	0.42 ± 0.21 (n = 35)	0.76 ± 0.29 (n = 19)	< 0.0001	0.811 (0.729 to 0.959)	> 0.45	89.5%; 77.1%	< 0.0001
Baseline 3D VCA, cm^2^; NT/NTR device (n = 30)	0.37 ± 0.13 (n = 21)	0.58 ± 0.22 (n = 9)	0.002	0.802 (0.602 to 1.000)	> 0.45	77.8%; 85.7%	0.0031
Baseline 3D VCA, cm^2^; XTR device (n = 24)	0.50 ± 0.27 (n = 14)	0.92 ± 0.26 (n = 10)	0.001	0.868 (0.719 to 1.000)	> 0.54	100%; 71.4%	< 0.0001

*3D* 3-dimensional, *CI* confidence interval, *TMVR* transcutaneous mitral valve repair, *VCA* vena contracta area

Postprocedural valve stenosis was defined as TMPG ≥ 5 mmHg whereas TMPG < 5 mmHg indicated a favorable result without relevant valve stenosis after TMVR. Baseline MVA assessed by 3D TEE was identified to be a predictor for postprocedural valve stenosis with different cut-off values for the NT/NTR device (cut-off: MVA ≤ 4.2 cm^2^: AUC = 0.920, 95% CI 0.809 to 1.000; sensitivity 87.5%, specificity 86.4%) and the XTR device (cut-off: MVA ≤ 6.0 cm^2^: AUC = 0.865, 95% CI 0.664 to 1.000; sensitivity 100%, specificity 66.7%) (Table 5).

**Table 5:** Impact of baseline mitral valve opening area (MVA) and mean transmitral pressure gradient (TMPG) on postprocedural valve stenosis

	TMPG < 5 mmHg after TMVR	TMPG ≥ 5 mmHg after TMVR	p value	AUC (95%-CI)	Cut-off value	Sensitivity; specificity	p value
Baseline MVA, cm^2^; all patients (n = 54)	6.4 ± 1.9 (n = 43)	3.9 ± 1.2 (n = 11)	< 0.0001	0.888 (0.789 to 0.987)	≤ 4.8	90.9%; 72.1%	< 0.0001
Baseline TMPG, mmHg; all patients (n = 54)	1.6 ± 0.7 (n = 43)	2.9 ± 1.0 (n = 11)	< 0.0001	0.877 (0.786 to 0.969)	> 1.9	100%; 65.1%	< 0.0001
Baseline MVA, cm^2^; NT/NTR device (n = 30)	5.7 ± 1.3 (n = 22)	3.4 ± 1.1 (n = 8)	< 0.0001	0.920 (0.809 to 1.000)	≤ 4.2	87.5%; 86.4%	< 0.0001
Baseline TMPG, mmHg; NT/NTR device (n = 30)	1.3 ± 0.5 (n = 22)	2.9 ± 0.8 (n = 8)	< 0.0001	0.949 (0.888 to 1.000)	> 1.9	100%; 72.7%	< 0.0001
Baseline MVA, cm^2^; XTR device (n = 24)	7.1 ± 2.1 (n = 21)	5.0 ± 0.9 (n = 3)	0.11	0.865 (0.664 to 1.000)	≤ 6.0	100%; 66.7%	0.0004
Baseline TMPG, mmHg; XTR device (n = 24)	1.8 ± 0.8 (n = 21)	3.1 ± 1.5 (n = 3)	0.03	0.794 (0.545 to 1.000)	> 1.8	100%; 57.1%	0.02

*AUC* area under the curve, *CI* confidence interval, *MVA* mitral valve diastolic opening area, *TMPG* mean transmitral pressure gradient, *TMVR* transcutaneous mitral valve repair

Accordingly, baseline TMPG has also shown to be a predictor for postprocedural valve stenosis with slightly different cut-off values for the NT/NTR device (cut-off: TMPG > 1.9 mmHg: AUC = 0.949, 95% CI 0.888 to 1.000; sensitivity 100%, specificity 72.7%) and the XTR device (cut-off: TMPG > 1.8 mmHg, AUC = 0.794, 95% CI 0.545 to 1.000; sensitivity 100%, specificity 57.1%) (Table 5).

## Discussion

The major findings of this study were:

1. the larger XTR device was equally effective for MR reduction assessed by 3D VCA although deployed in greater mitral valves and more severe MR compared to the NT/NTR device
2. distinct cut-off values for the NT/NTR device and the XTR device for prediction of successful TMVR with effective MR reduction and avoiding postprocedural valve stenosis with a single clip were identified
3. and, application of 3D echocardiography allowed detailed analysis of procedural effects of TMVR differentiated between the NT/NTR and the XTR device.

This is to our knowledge the first study to investigate procedural effects of the larger Mitraclip XTR device compared with the smaller NT or NTR device by detailed 3D echocardiographic analysis providing distinct cut-off values of successful TMVR for the different devices. Direct comparison of the XTR and NT/NTR group can be assumed to be more accurate than a comparison of one group to results of previous studies or older registries.

### Impact of clip size on MR reduction in different mitral valve anatomies

Since the beginnings of TMVR, patients suitable for TMVR have increased because of a rising experience and improved clip systems with different clip iterations. The clip strategy using one or more clips and the selection of clip size was left to the experienced judgement of the operator since no concrete exclusion parameters in the latest studies were defined [3, 4, 6]. This leads to a selection bias in our study: regarding mitral valve anatomy and MR severity, MitraClip XTR device was rather implanted in patients with longer mitral leaflets especially with longer posterior mitral leaflets, wider coaptation gaps leading to more severe MR and in patients with greater MVA, whereas the smaller MitraClip NT/NTR device was rather used in patients with shorter mitral leaflets, smaller MVA and already mildly elevated preprocedural TMPG. Concordantly, in the XTR group of this study MR was significantly more severe and MVA was greater. Nevertheless, reduction of MR was equally effective by XTR compared to NT/NTR devices with a trend in favor of the XTR Clip. There was no difference in relative reduction of MVA between the XTR and NT/NTR device. Despite the larger size of the XTR device, there was no difference in increase in postprocedural TMPG by the XTR device and the NT/NTR device (+ 1.0 ± 1.0 mmHg vs. + 1.7 ± 1.4 mmHg, p = 0.05).

In all NT/NTR patients and 96% of XTR patients MR reduction to mild-to moderate (2+) or less could be achieved according to 2D echocardiographic classification, what is comparable to a previous study from Praz et al. (2019), where the MitraClip XTR device was implanted successfully in 93% of the cases and achieved even a better reduction of MR in comparison to results of previous studies using smaller clips. In our study 24 patients were treated successfully with a single XTR-clip strategy. Praz et al. (2019) demonstrated that 57% of patients could be treated with a single clip strategy in their study and this amount of patients was considerably higher compared to 37% to 48% in previous studies in which only the smaller MitraClip device was used [3–5]. However, like in our study, Praz et al. presented that the use of the larger XTR device did not result in an increase of TMPG after TMVR (3.5 ± 1.8 mmHg) in comparison to older studies except for the combination NTR/XTR devices (4.4 ± 1.6 mmHg) [5].

Concordantly to previous studies, in case of suitable mitral valve anatomy a one clip strategy with the larger XTR device leads to an equally effective reduction of MR in larger mitral valves with more severe baseline MR in comparison to the smaller NT/NTR device.

### Prediction of successful TMVR based on baseline echocardiographic parameters

In our study different predictors of acute favorable outcome after TMVR could be identified. Baseline 3D VCA was a predictor of successful regurgitation reduction in a one clip strategy in secondary MR: distinct cut-off values with baseline 3D VCA > 0.45 cm^2^ in the NT/NTR device and > 0.54 cm^2^ in the XTR device were predictors of ineffective MR reduction as defined as postprocedural VCA > 0.2 cm^2^ after TMVR. In cases with baseline 3D VCA above that cut-off values, the selection of the larger XTR instead of the smaller device or a two or more clip strategy might have to be discussed.

A previous study in our institution using only the smaller MitraClip NT device demonstrated that in patients with a reduction of 3D VCA > 50% preprocedural mitral annulus area was smaller compared to patients with a reduction of 3D VCA ≤ 50% (11.9 ± 3.9 cm^2^ vs. 16.1 ± 8.5 cm^2^) [10]. In this study, concordantly preprocedural MVA was even slightly smaller with 11.4 ± 2.4 cm^2^ in patients receiving the XTR device and 9.9 ± 2.9 cm^2^ in patients receiving the NT/NTR device with relative reduction of 3D VCA of 60.6% in the NT/NTR group. This may lead to the assumption, that a one clip strategy might be especially successful in smaller mitral annulus areas. Further studies will be needed to compare different strategies using different amounts of clips and different clip sizes. Moreover, that previous study demonstrated that TMVR using the small MitraClip device in patients with baseline MVA < 4.1 cm^2^ by 3D echocardiographic assessment resulted in an elevated TMPG ≥ 5 mmHg most likely corresponding to moderate valve stenosis [10]. That result was consistent to the results of this present study in which a cut-off value of ≤ 4.2 cm^2^ for baseline MVA was found to predict postprocedural TMPG ≥ 5 mmHg using the NT or NTR device. A new finding in this study was that for the larger XTR device, a particular cut-off value of baseline MVA ≤ 6.0 cm^2^ assessed by 3D TEE was found to predict postprocedural TMPG ≥ 5 mmHg. Another predictor for a relevant postprocedural mitral stenosis as defined as postprocedural mean transmitral pressure gradient ≥ 5 mmHg was the baseline TMPG: in NT/NTR and XTR patients with baseline TMPG > 1.9 mmHg and respectively > 1.8 mmHg relevant mitral stenosis was seen after TMVR. In a previous study Alessandrini et al. underscore the importance of a preprocedural assessment by detailed echocardiography: the baseline TMPG in addition to other echocardiographic parameters were used to develop a nomogramm to predict the postprocedural TMP [11].

Our data suggest that preprocedural 3D TEE assessment is better suited for planning the best clip strategy and for correct selection of clip size to achieve a successful MR reduction without causing relevant mitral stenosis. Prospective studies will be needed to further investigate this suggestion and to evaluate long-term clinical outcome. Since one can choose between different edge-to-edge repair systems and different clip sizes, pre- and intraprocedural 3D assessment of mitral valve and mitral annulus geometry might become more important for planning the best TMVR approach.

## Limitations

The main limitations are that this study was monocentric and based on retrospective analyses of TMVR with the NT or NTR device and the XTR device and that only acute procedural effects of TMVR were evaluated. Long-term prognostic data have not been studied to assess the clinical impact of the results on patient outcomes. Then, there was a patient selection bias: mitral valve anatomy determines clip selection, so that the MitraClip XTR was suggested for greater valves with longer leaflets. The choice of the deployed device was left to the judgment of the operator. Furthermore, only patients who underwent TMVR with one clip were included into this study resulting in a considerable bias of patient selection. Decision of using not more than one clip was also based on the operators’ choice. Effects of TMVR with a two or more clip strategy using different combinations of clip sizes would be of interest, but analysis of these different groups might be complex as the different clips may interfere with each other depending on baseline mitral valve anatomy as well as heterogeneous deployment positions.

## Conclusions

3D TEE assessment allowed detailed analysis of procedural effects of TMVR in patients with secondary MR with different MitraClip devices. TMVR using the larger XTR device resulted in an equally effective reduction of MR despite of a greater baseline regurgitation severity compared to the smaller NT or NTR device without difference of postprocedural TMPG. Distinct baseline 3D VCA cut-off values were identified for prediction of acute successful MR reduction with the NT/NTR device and the XTR device in patients undergoing TMVR with one clip. Accordingly, particular cut-off values of baseline MVA and baseline transmitral pressure gradient for prediction of relevant increase of postprocedural TMPG were found for the NT/NTR device and the XTR device.

## Abbreviations

AML: Anterior mitral leaflet
EROA: Effective regurgitant orifice area
MR: Mitral regurgitation
MVA: Mitral valve area
PISA: Proximal isovelocity surface area
PML: Posterior mitral leaflet
RVol: Regurgitant volume
TEE: Transesophageal echocardiography
TMPG: Transmitral pressure gradient
TMVR: Transcatheter mitral valve repair
TTE: Transthoracic echocardiography
VCA: Vena contracta area
